# Editorial: Physical activity, health equity and health-related outcomes, volume II

**DOI:** 10.3389/fpubh.2024.1379960

**Published:** 2024-02-16

**Authors:** Ahmad Alkhatib, Noël C. Barengo

**Affiliations:** ^1^College of Life Sciences, Birmingham City University, Birmingham, United Kingdom; ^2^Herbert Wertheim College of Medicine, Florida International University, Miami, FL, United States

**Keywords:** health equality, reducing disparities, exercise and lifestyle modification, sport participation, public health interventions, children and adolescents, special population groups, health related quality of life

Despite the established benefits of physical activity (PA) in the prevention and treatment of disease, most population groups continue to suffer from the burden of non-communicable diseases (NCDs) including diabetes, cancer, and cardiovascular disease (1). Therefore, research into reducing health inequality and improving quality of life through promoting PA and associated healthy lifestyle behavior, is essential to impact public health policies and practice.

This Research Topic, “*Physical activity, health equity and health-related outcomes, Volume II*” (https://www.frontiersin.org/research-topics/28849/physical-activity-health-equity-and-health-related-outcomes-volume-ii/magazine), extends the findings of Volume-I (2). This Research Topic investigates emerging research on PA and disease epidemiology in special populations, which includes novel determinants of PA in a health equity and outcomes context, as well as effective PA-related intervention approaches amongst under-represented groups, including women, certain racial and ethnic groups, older adults, and children with poor PA and healthcare access. Publications in this Research Topic made an immediate impact, with 25,000 article views and 17,000 full-text views, and over 6,000 downloads. Out of the 41 submissions, we publish 13 high-quality manuscripts following rigorous editing and peer-reviewing. Research types varied from countrywide epidemiological surveys in Asia, Europe and the USA, to cross-sectional data analysis of longitudinal population surveys, PA and lifestyle determinants in children and adolescents, predictive tools, clinical and randomized controlled trials involving patients and high-risk populations.

Children and adolescents are considered as high-risk groups due to growing concern about childhood obesity and associated comorbidities, with over 2.1 billion worldwide (3). Alarmingly, there is an increased disparity in such prevalence between Low and Middle Income Countries (LMICs) and High Income Countries (HICs). Our team published a systematic review (Obita and Alkhatib) which involved 651,659 children, and showed a significantly higher prevalence of childhood obesity comorbidities in LMICs than in HICs (hypertension 36 vs. 13%, metabolic syndrome 27 vs. 6%, non-alcoholic fatty liver disease 48 vs. 23% in LMICs than HICs, respectively). Further disparity was found between global regions, where Asia had the highest prevalence of childhood obesity-related hypertension (38.6%) followed by South America (25.3%) and Europe (20.1%). Within HICs disparity was also found, where minority ethnic communities had higher childhood obesity and comorbidities than their white counterparts, especially in the prevalence of cardiometabolic risks of insulin resistance, dyslipidaemia and acanthosis. Global and local public health policies are needed to reduce disparity in childhood obesity and associated comorbidities through effective targeted interventions.

Explaining childhood obesity and comorbidity disparity requires deciphering various underlying lifestyle health behaviors by socioeconomic status (SES). A systematic review by Gautam et al. showed that children and adolescents with lower SES face an elevated risk of unhealthy behaviors such as initiation of smoking, energy-dense foods, lower PA, and drug abuse compared with their counterparts. Conversely, those from higher SES showed a higher prevalence of health-promoting behaviors including increased consumption of fruit and vegetables, dairy products, regular breakfast, and engagement in PA. These findings emphasize the need for interventions aimed at supporting families from disadvantaged SES to mitigate lifestyle disparities in health behavior among children and adolescents. For example, Shi et al. showed that promoting PA and sports participation, and muscle strengthening in schools is more effective than active commuting to schools. Their cross-sectional study involved 3,807 children and adolescents from 12 schools in South-eastern China. This finding is in line with recent preventative lifestyle models for children and adolescents, encouraging direct and intense forms of sport participation alongside wider PA promotion (4). For those in the earlier childhood preschool years, direct parental caregiving has been shown to be essential for developing children's fundamental movement skills (FMS), enhancing their PA participation, and developing physical and cognitive fitness (Hu et al.). The latter cross-sectional study involved 698 boys and 628 girls, aged 4–6 years, and showed that direct parenting, especially for girls is superior to grandparenting in developing children's FMS and learning more complex physical and sporting activities.

Nonetheless, reducing disparity should be addressed across all age groups. A longitudinal analysis (2010–2018) from the Chinese General Social Survey reported SES disparities and inequality of mass sports participation among 4,940 residents in China (Dong et al.). A higher frequency of PA participation was found in urban residents and higher social classes than in rural residents and lower social classes. Public-sector jobs, high income and higher education levels were associated with higher PA participation, and older adults reported more motivation to exercise than the young. Ethnic minority status, alone or in conjunction with SES were also a determinant of mass sports participation. Population sports participation often serves government health preventative policy. Whether it is mass sports, PA or regular exercise, sport and health policies should reduce disparity and inequality in both access and participation in PA.

Access to PA was particularly important throughout the COVID-19 pandemic, and thus PA interventions using remote across different populations need to be explored further. Two interesting studies focused on this Research Topic in China and India. Zhang et al. published an analysis of the China Family Panel Survey of 24,000 adults in the period preceding COVID-19 to understand the functional mechanisms determining PA and internet use. They showed a positive correlation between internet use duration and PA and fitness. The researchers suggested that the mediating effect of psychological health risk perceptions was more important than that of social capital determining PA behaviors. In India, a clinical trial involving tele-yoga as an adjunct PA along with standard treatment was an effective method in reducing COVID-19 symptoms in hospitalized patients with mild and moderate COVID-19 (Majumdar et al.). Perhaps remote PA environments are effective for a controlled community (e.g., quarantine) or hospital environments, but psychological health mechanisms should be considered alongside other lifestyle behaviors and environments. One study reviewed the impact of the environment on PA engagement and consequent health benefits (Hernández et al.). The study concluded that environmental social programs (e.g., school building design, parks, bicycle paths, or community or workspaces) generate positive changes in increasing PA levels and associated healthy lifestyle behavior among children and adults.

Epidemiological analysis of country-wide data sets is also essential in understanding the underlying mechanisms of disease in high-risk groups, and to mitigate such risks through targeted interventions. In Lithuanian populations, two biomarkers of cardiovascular disease (visceral adiposity index, and atherogenic index of plasma), were associated with all-cause mortality risk in groups of middle-aged men and women (Tamosiunas et al.). The Afghanistan WHO-STEPS survey 2018, analyzed sedentariness among 3,956 adults using the Global Physical Activity Questionnaire (Pengpid et al.). They reported that almost 50% of Afghani populations as sedentary while one in four had both high sedentary behavior and low PA. Another study in Greece (Knappe et al.) reported on the physical fitness (based on maximal oxygen uptake) and mental wellbeing of 150 men (50% women) living in refugee camps and suffering from post-traumatic stress disorder (PSTD). One in four participants met the criteria of metabolic syndrome, and a third met the criteria of anxiety, insomnia and PSTD, which highlights the intervention needs in this vulnerable population group.

Finally, reducing disparities and improving health equity requires the design of culturally and environmentally appropriate lifestyle interventions to ensure effective implementation in communities (5). The study protocol (Greeven et al.) designed multilevel PA-based interventions in rural communities based on concepts of (1) Basic Psychological Needs mini-theory within Self-Determination Theory; (2) Biopsychosocial Model (3) Multilevel Research Framework from the National Institute on Minority Health and Health Disparities. Another study focussed on validating the PA tool as an effective method to engage young students in PA (Liu et al.). The authors validated a device combining heart rate variability and accelerometry (Firstbeat Bodyguard 2) to measure time spent at different intensity zones in free-living environments.

This Research Topic highlights diverse PA preventative roles across different age groups, ethnicities, genders and different environments and elucidates on important physiological, psychological and socioeconomic factors that determine the effectiveness of PA participation and applications. Such research efforts alongside public health policies will improve health and quality of life for all populations.

## Author contributions

AA: Writing—original draft, Writing—review & editing. NB: Writing—original draft, Writing—review & editing.
